# Surgical management of metastatic squamous cell carcinoma of the bladder: a case report and review of the literature

**DOI:** 10.3389/fonc.2026.1806454

**Published:** 2026-04-13

**Authors:** Michael Schwartz, Caitlin Hartman, Greta McClintock, Sarah Caulfield, Jaishree Jagirdar, Lara Harik, David Berkowitz, Felix Fernandez, Sonal Oza, Vikram Narayan, Jacqueline T. Brown

**Affiliations:** Emory University School of Medicine, Atlanta, GA, United States

**Keywords:** bladder, oligometastasis, squamous cell carcinoma, surgery, systemic therapy

## Abstract

Nonurothelial bladder cancer is rare, and there is no consensus regarding optimal systemic therapy. Even less well-defined is the role for surgical resection of oligometastatic sites that progress despite systemic therapy. Here, we describe the case of a 65-year-old woman who underwent radical cystectomy for locally advanced pure squamous cell carcinoma (SqCC) and subsequently developed two substantial metastatic lung lesions shortly after surgery. After her disease did not respond to platinum-based doublet chemotherapy and immunotherapy, hospice was considered, given her declining performance status. However, she underwent a pulmonary bilobectomy, which resulted in a durable complete response lasting over 2 years. This case demonstrates a potential role for surgical intervention in the management of metastatic SqCC of the bladder.

## Highlights

- Bladder squamous cell carcinoma is a rare, aggressive, and less chemosensitive subtype of nonurothelial bladder cancer.
- Beyond radical cystectomy for primary disease, little is known regarding the management of bladder SqCC, particularly in the advanced stage.
- This report describes the successful management of metastatic bladder SqCC through surgical resection of metastases with long-term recurrence-free survival following progression through systemic therapy.
- Novel targeted therapies approved for use in urothelial carcinoma may provide additional options with which to treat nonurothelial bladder malignancies.

## Introduction

Bladder cancer represents a spectrum of disease with various histologic subtypes. Urothelial carcinoma (UC) is the most common subtype, representing approximately 90% of bladder cancer diagnoses (1). Due to its prevalence, significant progress has been made in the management of localized and advanced UC (2). Nonurothelial carcinomas represent less than 10% of bladder cancer diagnoses and include squamous cell carcinoma (SqCC), adenocarcinoma, and other rarer carcinomas such as small cell high-grade neuroendocrine carcinoma (3). Due to their relative rarity and the common exclusion of these nonurothelial subtypes from clinical trials, limited data are available to guide their treatment (4).

Pure squamous cell carcinoma is the most common non-UC bladder malignancy, representing 2%–7% of all bladder cancers (4). Compared with UC, SqCC generally has a more aggressive course and is less chemosensitive (1, 5). For these reasons, localized SqCC of the bladder is primarily treated with early radical cystectomy or pelvic exenteration without neoadjuvant or adjuvant chemotherapy (NAC) (6–8). Similarly, the management of *metastatic* SqCC remains largely unexplored. Treatment is often extrapolated from paradigms for SqCC originating from nonbladder organs and is managed on a case-by-case basis (9). Here, we describe the management of metastatic squamous cell carcinoma of the bladder in which aggressive surgical resection resulted in a durable complete response after the disease was poorly responsive to systemic therapy.

## Case

**Figure 1:**
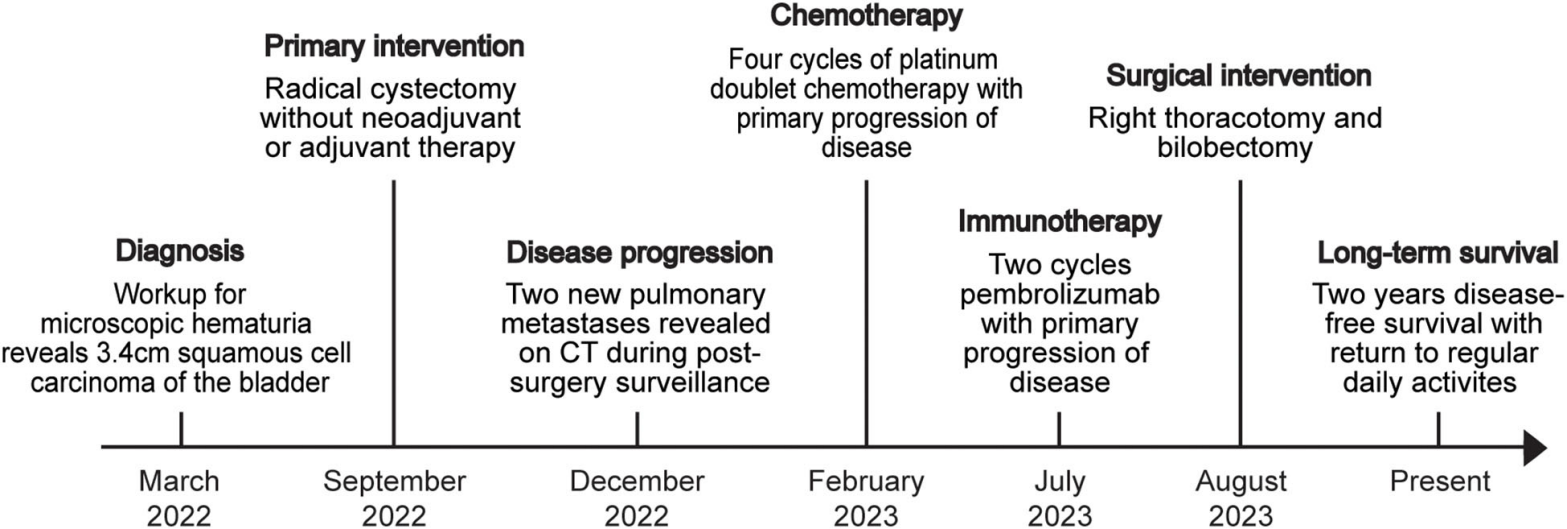
Clinical timeline.

**Figure 2:**
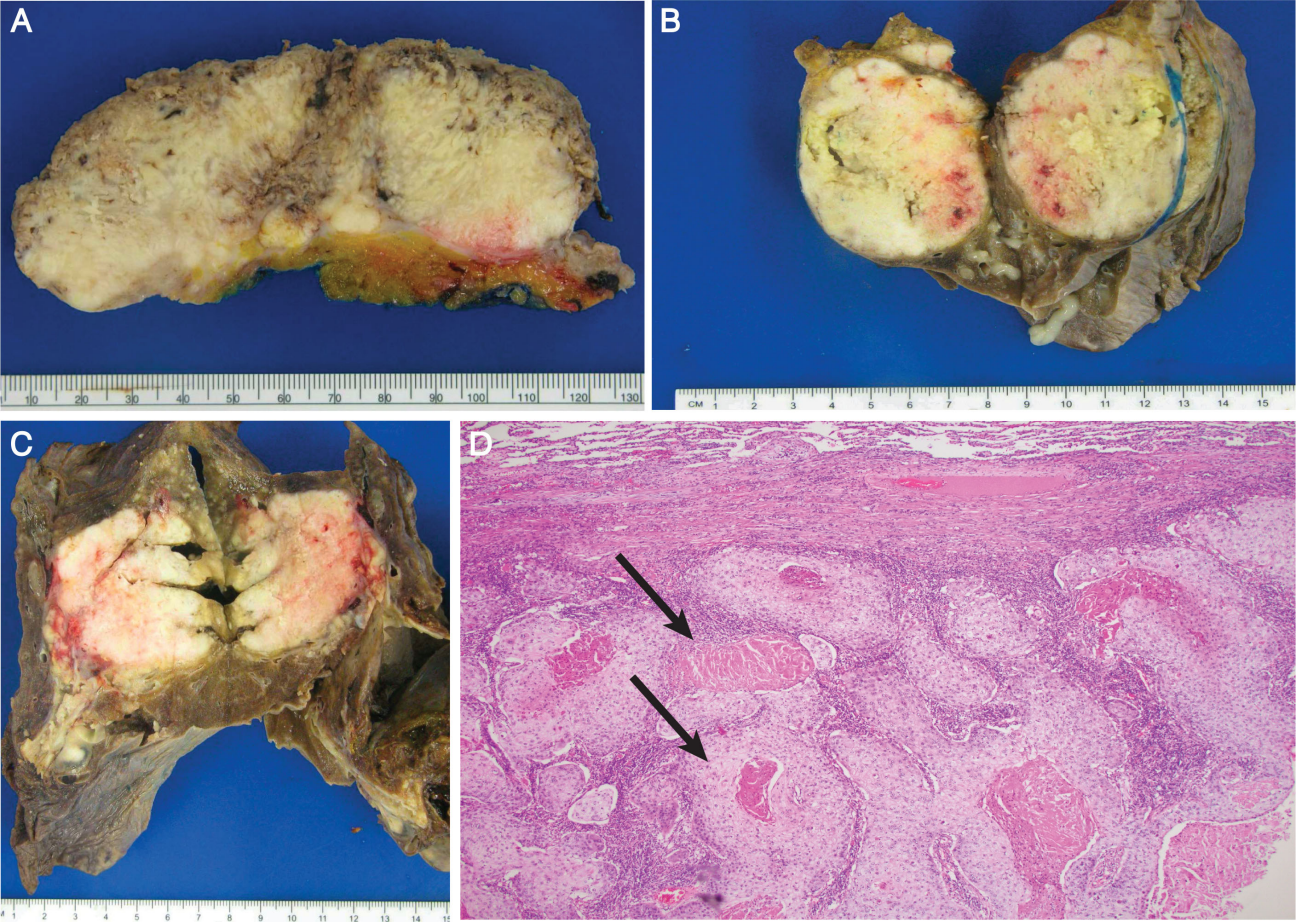
Gross pathology specimen from cystectomy showing an 11.9-cm white friable mass extending grossly into perivesical fat **(A)**. Resection specimen from pulmonary bilobectomy of the middle lobe **(B)** and lower lobe **(C)** pulmonary masses, confirmed microscopically to be squamous cell carcinoma with characteristic keratinizing pearls **(D)**.

A 65-year-old woman presented with microscopic hematuria and was referred to urology (**Figure 1**). Cystoscopy revealed a 3.4-cm bladder mass, and subsequent transurethral resection of bladder tumor (TURBT) revealed a muscle-invasive, high-grade neoplasm with squamous morphology. Computed tomography (CT) showed a fungating mass originating from the lateral bladder wall and filling the bladder lumen. CT of her chest did not show evidence of metastatic disease. She underwent radical cystectomy with ileal conduit and pelvic lymphadenectomy. Final pathology of the tumor revealed an 11.9 cm × 10.0 cm × 4.4 cm pure, moderately differentiated keratinizing SqCC with necrosis (**Figure 2A**). Surgical margins were negative. Pelvic lymph nodes were dissected and uninvolved. Final pathologic stage was pT3bN0.

**Figure 3:**
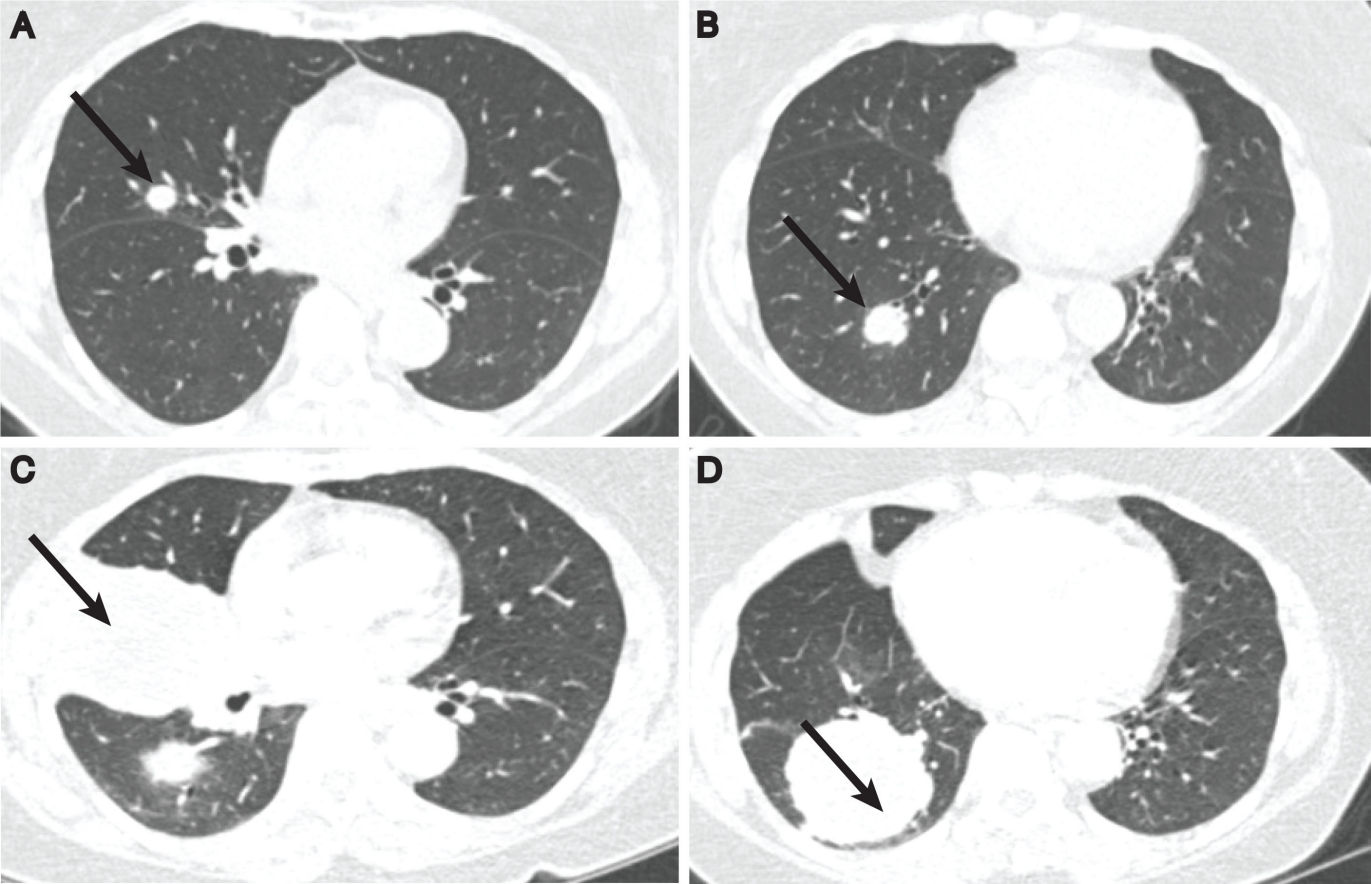
Chest CT imaging demonstrating pulmonary masses at the time of diagnosis in the right middle lobe **(A)** and the right lower lobe **(B)**. Despite platinum doublet chemotherapy and immunotherapy, imaging showed significant enlargement of the right middle lobe **(C)** and right lower lobe **(D)** lesions prior to her bilobectomy.

The patient was referred to medical oncology to discuss postoperative management. She underwent somatic next-generation sequencing (NGS) in an attempt to inform adjuvant therapy. NGS revealed that her tumor was negative for programmed death ligand 1 (PD-L1), with low tumor mutational burden (TMB) and no other actionable mutations. A decision was made to pursue surveillance. Unfortunately, cross-sectional imaging 3 months after surgery showed two new pulmonary lesions, including a 1.3-cm mass in the right middle lobe and a 2.2-cm mass in the right lower lobe (**Figures 3A, B**). She underwent bronchoscopy and transbronchial biopsy of both lesions. Pathology was consistent with metastatic SqCC of bladder origin. Treatment with cisplatin and paclitaxel was initiated. The patient developed significant peripheral neuropathy after two cycles, necessitating a switch from cisplatin to carboplatin. Imaging after four total cycles of chemotherapy showed a mixed response to therapy, with an increase in the right middle lobe mass and a decrease in the right lower lobe mass. Her case was discussed at the multidisciplinary tumor board, at which time thoracic surgery was considered palliative in intent with significant morbidity; consensus was to continue systemic therapy.

She received two additional cycles of carboplatin and paclitaxel, given a paucity of other systemic options, totaling six cycles of chemotherapy. Pembrolizumab therapy was initiated soon after as a maintenance strategy, and two cycles were given. Subsequent imaging showed growth of both pulmonary lesions, causing mass effect on the bronchi and abutting the pleura without underlying bony erosion to suggest osseous extension. A slight enlargement of several lymph nodes was noted, including a 1.6 cm × 1.0 cm left hilar lymph node and a 1.8 cm × 0.8 cm right lower paratracheal lymph node, previously 0.4 cm and 0.9 cm × 0.5 cm, respectively (**Figures 3C, D**). She was starting to experience shortness of breath, increasing fatigue, and scant hemoptysis. After urgent consultation with thoracic surgery, it was decided that surgical resection would be pursued with palliative intent and significant potential risk to her respiratory capacity.

Within 1 month, she underwent a right thoracotomy, right middle (**Figure 2B**) and right lower lobe (**Figure 2C**) bilobectomy, and mediastinal lymphadenectomy. The operation was uncomplicated, and she was hospitalized for 5 days. Final pathology showed two large SqCC tumors measuring 9.7 cm and 7.1 cm, microscopically consistent with metastatic squamous cell carcinoma (**Figure 2D**). Both tumors showed more than 50% viable tumor. Surgical margins were negative, the pleura was uninvolved, and one of 19 lymph nodes was involved with metastatic squamous cell carcinoma. All other lymph nodes showed evidence of noncaseating granulomas consistent with sarcoidosis. She remains without evidence of recurrence 2 years after surgical resection. She has recovered from surgery, has no residual sensory peripheral neuropathy following consultation and treatment with physiatry, and remains active, playing pickleball several times weekly and writing her first children’s book.

## Discussion

The treatment frontier in urothelial bladder cancer has advanced significantly in recent years with the approval of enfortumab vedotin (EV) and pembrolizumab in the first-line setting, regardless of cisplatin eligibility. This combination set a new benchmark for survival at 31.5 months, resulting in a very dynamic later-line setting as clinicians are left deciding how to sequence platinum-based chemotherapy, Fibroblast Growth Factor Receptor (FGFR)-directed therapy in those with a susceptible FGFR3 alteration, and novel agents being investigated in clinical trials (10). Ongoing advances in antibody–drug conjugate (ADC) therapies are of special interest in advanced UC, with fam-trastuzumab deruxtecan-nxki now approved for advanced UC with high Human Epidermal Growth Factor Receptor 2 (HER2) expression and other ADCs like disitamab vedotin showing promise (11, 12). In contrast, advancement in nonurothelial bladder cancer has been stagnant due to the rarity of the disease and a lack of prospective clinical trials. As the most common nonurothelial bladder cancer, SqCC presents particular challenges in its management.

Neoadjuvant and adjuvant therapies have shown little evidence of efficacy in the management of SqCC; therefore, early radical cystectomy alone is the standard of care for local disease. In one retrospective study of 671 patients with T2–T3 SqCC investigating the use of NAC before radical cystectomy (RC), no benefit in overall survival (OS) was identified in the NAC group (5). A second retrospective study of 2,018 patients with T2 muscle-invasive bladder cancer with variant histologies, including 810 patients with SqCC, showed no OS benefit with NAC in the SqCC group (13). Additionally, a meta-analysis of seven studies found no benefit in cancer-free survival in patients with SqCC receiving NAC (14). Regarding the use of adjuvant chemotherapy, few studies have explored its role in bladder cancers with variant histologies. One meta-analysis of four studies that reported survival outcomes in SqCC treated with RC and adjuvant chemotherapy found no associated benefit in recurrence-free survival or OS (14). While adjuvant nivolumab is considered standard of care for patients with high-risk residual urothelial carcinoma at the time of cystectomy, it is important to note that the CHECKMATE274 study did not allow patients with nonurothelial histologies like SqCC or even UC with significant divergent differentiation (15). Some studies, on the other hand, have found adjuvant radiation therapy (RT) to be an effective management strategy in patients with SqCC following RC. *Post hoc* analysis of a phase III randomized trial comparing adjuvant RT, adjuvant RT plus chemotherapy, and adjuvant chemotherapy alone in 198 patients with locally advanced bladder cancer identified improved OS in the RT group vs. the chemotherapy-only group (16, 17). The lack of strong evidence supporting adjuvant treatment in patients with SqCC following RC supported the decision not to provide adjuvant therapy to our patient following surgery.

In the metastatic SqCC setting, very few data are available to guide treatment. While SqCC typically presents at later stages as a result of its more aggressive phenotype, it is less likely to metastasize than UC, and the major cause of mortality in SqCC patients is local recurrence (9). Large-scale studies specifically investigating metastatic SqCC have not been possible, and case series and reports guide management. Platinum chemotherapy and checkpoint inhibition are mainstays in the management of the much more common squamous cell lung cancer, and oncologists often extrapolate from this body of literature, as we did for this patient. Insight into the effectiveness of chemotherapy or checkpoint inhibitor therapy (CPI) in advanced SqCC of the bladder specifically shows limited and often conflicting evidence. Banek et al. described the cases of two patients who received gemcitabine/cisplatin and one patient receiving nivolumab, none of whom responded to therapy (9). A prospective trial of ifosfamide, paclitaxel, and cisplatin in patients with advanced or metastatic non-UC bladder cancer showed an overall response rate of just 25% of the eight patients with SqCC (18). A phase II trial showed no response to PDL1 or CTLA4 inhibitors in 13 non-UC bladder cancer patients (three of whom had SqCC) (19). However, individual instances of response to CPI therapy do exist in the literature (20). In our patient, chemotherapy and CPI treatment were pursued due to a lack of alternatives, and they managed her disease ineffectively.

Biomarker identification in urothelial carcinoma has driven the development of novel therapeutics. Nectin-4, Trop-2, and HER2 are proteins known to be expressed and/or overexpressed in UC (21). Agents targeting these proteins have altered the field of advanced UC. A logical next step is whether data exists for their application to nonurothelial bladder cancers like SqCC. The rapid progression of this patient’s disease after chemotherapy and CPI forced the issue of surgery before alternative investigational agents could be explored.

EV is an antibody–drug conjugate that targets Nectin-4, a cell–cell adhesion protein expressed in UC, and is now a mainstay of treating first-line advanced UC in combination with pembrolizumab, based on EV-302, which showed superiority of the combination over platinum doublet chemotherapy (22). UC with squamous differentiation has been shown to have high Nectin-4 expression, so there is a rationale for EV efficacy in this variant (23). The UNITE study is a multi-institutional retrospective analysis of patients treated with EV and has included those with UC with divergent differentiation or non-UC disease altogether in addition to conventional UC (24). In patients with squamous differentiation of any kind (*n* = 94), the objective response rate (ORR) was 47%. In 17 patients where squamous differentiation was predominant amidst UC, the ORR was 33%. Only seven patients had pure SqCC with a disappointing ORR of 0% (25). More data are needed to determine the role of EV alone or in combination in treating UC with squamous differentiation or pure SqCC of the bladder. A phase II trial of EV and pembrolizumab in advanced or metastatic non-UC subtypes of bladder cancer is currently enrolling at our institution (NCT05756569).

Until recently, sacituzumab govitecan (SG), an ADC targeting Trop-2, was used in the standard later-line management of advanced UC based on accelerated FDA approval showing encouraging activity (ORR 27%) seen in the single-arm TROPHY-U-01 phase-II trial (26). Unfortunately, the confirmatory phase III study Tropics-04 was negative when comparing SG to single-agent chemotherapy in patients pretreated with platinum and CPI therapy (27). The future of SG in advanced UC hangs in the balance as a result. Trop-2 expression is present in SqCC and may represent a viable target in this population (28). Currently, a phase II trial exploring the use of neoadjuvant SG in patients who are cisplatin-ineligible prior to radical cystectomy is underway (NCT05581589).

HER2 is another target of interest in advanced UC. Trastuzumab deruxtecan was recently approved for any tumor with high HER2 expression (defined by immunohistochemistry (IHC) 3+) (12). Patients with UC (*n* = 41) in the phase II, single-arm, multicohort basket trial of this agent experienced an ORR of 39%; this increased to 56.3% in the patients with IHC 3 +. Other HER2-targeting agents like disitamab vedotin have shown promise in UC, both alone and in combination with CPI (11, 29). HER2 amplification is a more frequent finding in UC than in SqCC; one study demonstrated 82% of UC samples and only 6% of SqCC harbor HER2 amplification (30). With that said, given a lack of systemic alternatives, oncologists should investigate HER2 expression in tumors from patients with SqCC of the bladder in case this exploitable target is present. No trials are currently investigating the use of anti-HER2-targeted therapies in SqCC or UC with divergent histologies specifically.

The most remarkable aspect of this patient’s case is the demonstration of surgical efficacy in an oligometastatic disease state that developed metastases soon after curative-intent surgery and was unresponsive to systemic therapy. With lung metastases developing within 3 months of cystectomy, surgical resection was not immediately pursued, given the high likelihood of micrometastatic disease elsewhere (31). In recent years, the role of metastasectomy in managing patients with oligometastatic disease has garnered interest. Oligometastatic disease has been described as a distinct entity from its local and widely metastatic counterparts. There are differing opinions on its definition, but in general, it is an intermediate state in which the tumor has not yet developed the capacity to widely disseminate in the body and may still be susceptible to targeted and potentially curative local therapies (32, 33).

Local consolidative therapy (LCT) and metastasis-directed therapy (MDT) are two approaches to managing oligometastatic disease. LCT involves the treatment of remaining disease sites (primary or metastatic) after the receipt of primary systemic therapy, whereas MDT is the targeted treatment of metastatic sites regardless of whether systemic therapy has been given. In non-small cell lung cancer (NSCLC), the use of LCT has become integral to management in the oligometastatic state (34). A landmark study demonstrated that compared to observation alone, LCT in the form of surgery or RT in patients with oligometastatic NSCLC and stable disease following systemic therapy resulted in improved progression-free survival (PFS) (35). Mitchell et al. later reported that surgery imparted greater survival benefits compared with RT in this context (36). Phase III clinical trials continue to investigate the role of LCT in oligometastatic NSCLC (NCT03391869, NCT03707938). In prostate cancer, the WOLVERINE meta-analysis demonstrated that MDT improved PFS and castration-resistant-free survival, though overall survival benefit was not significant. Most patients in this analysis received RT as an MDT modality, with surgical resection selected on a case-by-case basis (37). Additionally, the phase II ORIOLE trial demonstrated improved survival in patients with oligometastatic prostate cancer whose metastatic sites were treated with stereotactic ablative radiotherapy (38). Similarly, the STOMP trial demonstrated prolonged androgen deprivation therapy-free survival in patients with oligometastatic prostate cancer treated with MDT (39). Currently, NCCN guidelines recommend MDT as *preferred* for metachronous oligorecurrent disease and oligometastatic castration-resistant prostate cancer and as a consideration for synchronous oligometastatic disease.

The role of MDT has been studied in bladder cancer as well. In a meta-analysis of patients with oligometastatic bladder cancer, MDT resulted in significantly prolonged survival. MDT modality varied, but surgical resection was specifically shown to be superior in several of the cited studies (40). In a separate study, Al-Nader et al. demonstrated improved survival with metastasectomy in patients with oligometastatic UC (41). Another study by Kim et al. demonstrated improved survival with metastasectomy in patients with urinary tract cancers and pulmonary metastases (42). A systematic review of surgical intervention in metastatic UC suggests that resection of local lymph node metastases or pulmonary metastases in oligometastatic disease may provide survival benefit in select patients, but primarily those with a measurable response to NAC (43). These studies are limited by small sample sizes; together, they indicate that surgical resection of metastases may be reasonable across numerous cancer types in the oligometastatic setting.

With respect to oligometastatic SqCC of the bladder, very little data exist regarding surgical management. In fact, studies investigating any form of LCT or MDT for metastatic nonurothelial carcinoma are, in general, extremely limited. In one case report, a 66-year-old man with prior RC for UC with significant squamous differentiation presented with an isolated metastasis in the right upper lobe of the lung. Right upper lobectomy with no adjuvant treatment resulted in at least 27 months with no evidence of recurrence or progression (44). The decision for surgery in our patient was fraught with multidisciplinary concern that her progressive micrometastatic disease would be incurable despite her bilobectomy. Additionally, most data to support LCT or MDT are in patients who have achieved a significant response to systemic therapy; our patient was different. The theoretical benefit of LCT or MDT was unclear at the time of clinical decision-making, given the progression of her disease throughout systemic therapy. Surgery was in part rationalized because of its potential to alleviate her local symptoms and stabilize her rapidly growing pulmonary disease, such that she would theoretically have the opportunity to pursue further systemic therapy. Her 2 years of disease-free survival thereafter, without the need for further systemic therapy, were unexpected and warranted this case presentation of the potential viability of surgical resection of metastatic disease in pure SqCC of the bladder in a patient with rapid relapse after cystectomy and primary resistance to systemic therapy.

## Data Availability

The original contributions presented in the study are included in the article/supplementary material. Further inquiries can be directed to the corresponding author.
